# Electrically Tunable Left-Handed Textile Metamaterial for Microwave Applications

**DOI:** 10.3390/ma14051274

**Published:** 2021-03-08

**Authors:** Kabir Hossain, Thennarasan Sabapathy, Muzammil Jusoh, Ping Jack Soh, Mohd Haizal Jamaluddin, Samir Salem Al-Bawri, Mohamed Nasrun Osman, R. Badlishah Ahmad, Hasliza A. Rahim, Mohd Najib Mohd Yasin, Nitin Saluja

**Affiliations:** 1 Advanced Communication Engineering (ACE), Centre of Excellence, Universiti Malaysia Perlis (UniMAP), Jalan Tiga, Pengkalan Jaya Business Centre, Kangar 01000, Malaysia; hossain.kabir42@gmail.com (K.H.); muzammil@unimap.edu.my (M.J.); pjsoh@unimap.edu.my (P.J.S.); nasrun@unimap.edu.my (M.N.O.); haslizarahim@unimap.edu.my (H.A.R.); najibyasin@unimap.edu.my (M.N.M.Y.); 2 Faculty of Electronic Engineering Technology, Kampus Alam UniMAP Pauh Putra, Universiti Malaysia Perlis (UniMAP), Arau 02600, Malaysia; badli@unimap.edu.my; 3 Wireless Communication Centre, Faculty of Electrical Engineering, Universiti Teknologi Malaysia, Johor Bahru 81310, Malaysia; 4 Space Science Centre, Climate Change Institute, Universiti Kebangsaan Malaysia, Bangi 43600, Malaysia; s.albawri@gmail.com; 5 Centre of Excellence Advance Computing (AdvComp), Kampus Alam UniMAP Pauh Putra, Universiti Malaysia Perlis (UniMAP), Arau 02600, Malaysia; 6 Chitkara University Institute of Engineering and Technology, Chitkara University, Punjab 140401, India; nitin.saluja@chitkara.edu.in

**Keywords:** tunable metamaterials, textile metamaterial, reconfigurable structure, DNG metamaterials, antenna and propagation

## Abstract

An electrically tunable, textile-based metamaterial (MTM) is presented in this work. The proposed MTM unit cell consists of a decagonal-shaped split-ring resonator and a slotted ground plane integrated with RF varactor diodes. The characteristics of the proposed MTM were first studied independently using a single unit cell, prior to different array combinations consisting of 1 × 2, 2 × 1, and 2 × 2 unit cells. Experimental validation was conducted for the fabricated 2 × 2 unit cell array format. The proposed tunable MTM array exhibits tunable left-handed characteristics for both simulation and measurement from 2.71 to 5.51 GHz and provides a tunable transmission coefficient of the MTM. Besides the left-handed properties within the frequency of interest (from 1 to 15 GHz), the proposed MTM also exhibits negative permittivity and permeability from 8.54 to 10.82 GHz and from 10.6 to 13.78 GHz, respectively. The proposed tunable MTM could operate in a dynamic mode using a feedback system for different microwave wearable applications.

## 1. Introduction

Metamaterials (MTMs) are artificially engineered materials featuring flexibility in their electromagnetic (EM) properties [1]. MTMs can be categorised as single-negative (SNG) or double-negative (DNG)/left-handed (LH) MTM, depending on the values of dielectric permittivity (*ε*) or magnetic permeability (*μ*). If either one of these properties is negative, the MTM is known as a SNG MTM. The SNG MTM with negative *ε* is known as the epsilon negative (ENG) MTM and SNG MTM with negative *μ* is known as mu-negative (MNG) MTM. Finally, the MTM with both negative permittivity and permeability is known as DNG/LH MTM [2,3]. The degree of reflection and refraction in a material can be calculated using the refractive index of a material based on its permittivity and permeability [1].

The most widely used MTM designs are based on arrays of compact resonators. They can be integrated with antennas to produce customised reactions and mode tuning. Different methods have been developed and are either based on loops (magnetic resonators), wires (electric), and their combinations [4,5]. Besides, researchers have employed MTMs in applications such as invisibility cloaking [6,7], wireless health monitoring [8], filters [9], sensors [10], bendable artificial magnetic conductors (AMC) [11], radio-frequency identification (RFID) tags [12], and electromagnetic wave absorbers [13]. In addition to this, MTMs have been used in textile-based wireless body area network (WBAN) antennas to minimise coupling between the antenna and the human body [14].

However, most MTM-based devices are static, i.e., upon completion of their fabrication, their EM properties cannot be controlled. Thus, the ability to control its material properties (i.e., *ε*, *μ*) is a highly desirable property in MTMs to enable their wider application. Moreover, technology requirements in communication engineering have increased the need for reconfigurable and multifunctional systems [15]. This has also increased the interest in researching tunable/reconfigurable MTMs. Reconfigurable/tunable MTM structures can overcome most of the significant limitations of static MTMs (i.e., high losses, narrow bandwidth (BW), and tolerance sensitivity) [16].

Material properties can be regulated using methods such as mechanical deformation and electrical control (e.g., varactors, switches), which tend to be the preferred approach [16]. Besides being low cost, varactor diodes are popular instead of expensive phase-shifting devices in beam steering applications [17]. These electrically driven components (e.g., varactors) can be regulated using external voltages or currents. Such a tunable method also immensely benefits the tunability of two-dimensional MTMs (also known as metasurfaces). This has resulted in the development of many practical devices such as low-profile and horn antennas, enabling the manipulation of their radiation patterns [15,18]. In addition, the control of refraction can be performed on-demand [15,19]. In most of the existing literature, varactor diodes have been used for single parameter tuning (i.e., either MNG properties or transmission coefficient). An example is the tunable MTMs implemented in absorbers [19] with a controllable absorption level depending on the applied reverse bias voltage. The varactor is substituted with an equivalent capacitance, thus, a full DC biasing circuit to practically tune the MTM is not available. Another work with a similar lack of DC biasing information is presented in [17], where a varactor diode was also used to tune the refractive index of the MTM unit cells. Despite this, practical DC biasing circuits were not fully implemented in both previous works. Furthermore, tunable MTMs, which can simultaneously reconfigure multiple parameters to produce different configurations of ENGs, MNGs and a refractive index are non-existent in the literature. For instance, a reflection coefficient tunability feature was achieved in [20], whereas [15] proposed material with tunable MNG properties.

Numerous studies have been performed over the past decade on methods to incorporate sensors and smart devices onto the human body more easily [21]. For this purpose, textiles have been widely chosen in wearable electronic applications as they are a comfortable alternative as substrate. The recent miniaturisation of electronic components and the introduction of new technologies have allowed electronic functionality to be incorporated into fabrics [22,23]. Recent literature has documented the development of fabrics, e.g., sewn textile materials, embroidered fabric material, nonwoven textiles, woven fabrics, knitted fabrics, braiding, spinning fabrics, printed fabrics, laminated fabrics, and chemically treated fabrics [24]. These materials have been developed to be incorporated as textile-based sensors in recent years. Many of the studies have concentrated on areas such as emergency rescue services and law enforcement [25], athletic training [26], fitness monitoring [27]. Fundamental aspects in designing reconfigurable/tunable MTMs for metamaterial-enhanced devices include fabrication complexity due to additional DC biasing [16], whereas additional considerations must be accounted for when designing textile-based MTMs early in their design stage [24].

This paper proposes a flexible, electrically tunable, multiparameter textile-based MTM that is the first of its kind, to the best of our knowledge. It was designed based on a decagonal-shaped split-ring resonator (SRR) and a slotted ground plane loaded with varactors. Simultaneous multiparameter tunability was enabled in the unit cells using RF varactor diodes. The proposed MTM was first designed and investigated independently prior to its implementation in 1 × 2, 2 × 1 and 2 × 2 arrays of unit cells. To extract the parameters of the MTMs, the robust reflection-transmission (RTR) method was adopted. The 2 × 2 array structure was then chosen to be experimentally validated using the waveguide (WG) port measurement technique [28]. Simulations and measurements showed tunable DNG characteristics within the range of 2.74 to 5.51 GHz. Simulations also showed that the MTM exhibited MNG and ENG properties within the range of 8.54 to 10.83 GHz and 10.63 to 13.79 GHz, respectively. Table 1 compares the proposed work against various reconfigurable/tunable MTMs found in the literature, indicating the MTM’s unique, flexible characteristics with multiparameter tuning capability.

## 2. Tunable Metamaterial Unit Cell Design

The proposed tunable MTM unit cell was designed, simulated and fabricated on flexible textiles. The SRR radiators and the slotted ground plane were designed using ShieldIt Super^TM^ conductive textile from LessEMF Inc., whereas felt was used as its substrate. The dielectric constant of felt is 1.44 and the loss tangent is 0.044. ShieldIt, on the other hand, has a conductivity of 1.18 × 10^5^ S/m and is 0.17 mm thick. The finite integration technique (FIT) in Computer Simulation Technology (CST)-Microwave Studio Suite (MWS) was used to simulate the MTM unit cell and the unit cell arrays.

**Figure 1:**
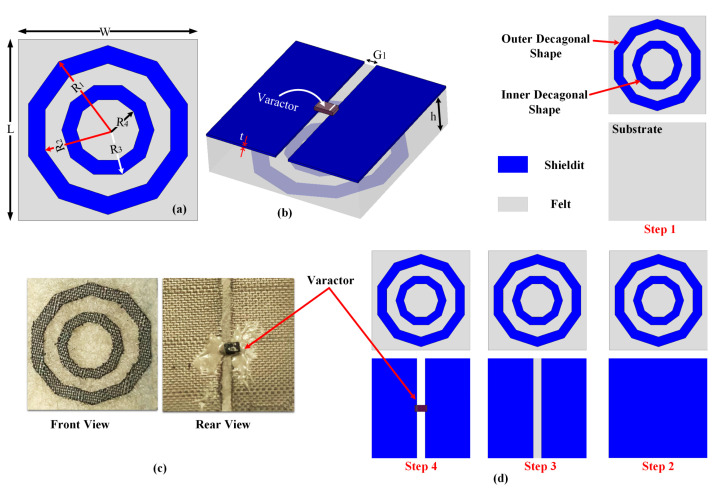
Schematic of the proposed unit cell: (**a**) Front view. (**b**) 3D back view. (**c**) Fabricated unit cell. (**d**) Design steps of the MTM unit cell.

**Figure 2:**
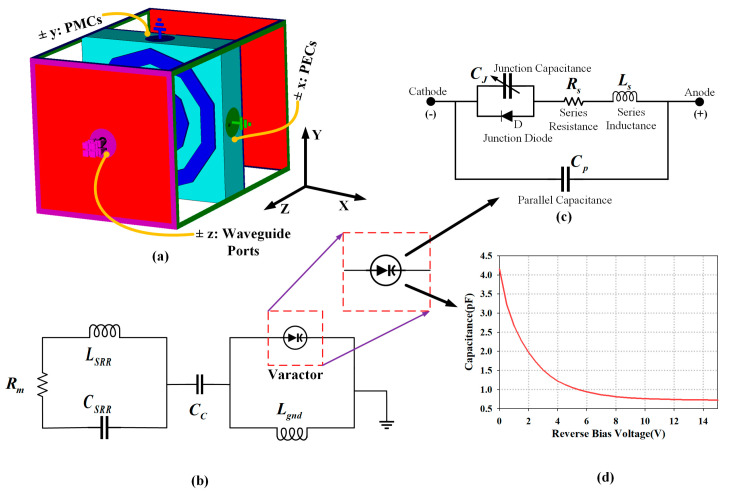
(**a**) 3D view of the electromagnetic (EM) MTM simulation setup for unit cell (1 × 1 array). (**b**) Equivalent circuit model of the proposed metamaterial unit cell. (**c**) Equivalent circuit model of the RF varactor diode (SMV1232-079LF from Skyworks, Inc) [31] and (**d**) relationship between the applied reverse bias voltage and total capacitance of the RF varactor.

Figure 1a,b depict the proposed MTM unit cell designed by combining a decagonal-shaped SRR resonator and a slotted ground plane. The RF varactor diode (SMV1232-079LF, Skyworks, Irvine, CA, USA) was placed across the slotted ground plane. The varactor’s capacitance was tuned based on the applied reverse bias voltage [31]. Figure 1c depicts the fabricated MTM unit cell. All physical parameters of the MTM unit cell design are summarised in Table 2. Based on its electromagnetic response, the decagonal-shaped SRR resonators behaved as an LC resonator circuit. That is, inductances are formed by the decagonal SRR, whereas the gap between two decagonal SRRs introduces a capacitance. Another coupling capacitance is introduced due to the gap between the metallic ground plane and the decagonal SRR. Finally, the ground plane adds to the inductance whereas the split on this ground plane introduces a capacitance. This capacitance can be altered by tuning the RF varactor. The detailed circuit is depicted in Figure 2b.

The overall design steps of the MTM structure, including modifying the ground plane to insert the RF varactor, are summarised in Figure 1d. First, the outer and inner decagonal-shaped splits were modelled. The next three steps completed the design procedure of the proposed MTM unit cell. In this study, a simple decagonal-shaped SRR was considered in the design phases to avoid fabrication complexity. This is due to the use of flexible textile-based materials and the need for DC biasing circuitry for the tuning of the material parameters. Besides, the use of such a decagonal SRR structure has been proven to enhance the magnetic resonance of the material [32].

## 3. Methodology

Figure 2a depicts the setup of the boundary conditions for the MTM in the simulations. Waveguide ports were defined along the *±z*-axis of the MTM unit cell. To excite the transverse electromagnetic (TEM) wave, a perfect electric conductor (PEC) boundary was defined at the ±*x*-axis whereas a perfect magnetic conductor (PMC) boundary was defined at the ±*y*-axis. The structure was meshed using tetrahedral meshes and simulated using the frequency domain solver available in CST MWS. Simulations were performed to independently characterise the properties of MTM unit cells from 1 to 15 GHz. This same setup was used to retrieve the material parameters for the 1 × 2, 2 × 1, and 2 × 2 unit cell arrays.

An equivalent circuit model of the MTM and the RF varactor diode is presented in Figure 2b, providing an insight into its working principle. The detailed equivalent circuit model of the RF varactor diode and other physical unit cell components are illustrated in Figure 2c, with its parameters defined as follows: *C_SRR_* is the capacitance across the gap between SRR rings, *L_SRR_* is the inductance of the SRR, *R_m_* is the dielectric and conductor losses, *C_c_* is the coupling capacitance between the metallic ground plane and the SRR, and *L_gnd_* is the inductance from the ground plane. To simplify the RF varactor model, the series resistance *R_S_* = 1.5, and series inductance *L_S_* = 0.7 nH were set as constants [31]. The effect of the junction diode was also considered negligible due to its limited operation of using only reverse bias voltage. The junction capacitance (*C_J_*) changes based on the applied reverse DC voltage (*V_R_*), and the relationship between them is described in Equation (1), as follows:

(1) $$C_{J}(V_{R})=\frac{C_{JO}}{{\left(1+\frac{V_{R}}{V_{J}}\right)}^{M}}$$

Note that in Equation (1), the parameters *C_JO_* = 4.2 pF, *M* = 0.9, and *V_J_* = 1.7 V are fixed. The total capacitance (*C_T_*) is the parallel combination of *C_J_* and the package capacitance (*C_P_*), as shown in Equation (2), with *C_P_* assumed to be 0 pF. When the reverse bias voltage is applied on the RF varactor, the junction capacitance of the varactor changes. The relationship between the reverse bias voltage and total capacitance is illustrated in Figure 2d.

(2) $$C_{T}(V_{R})=C_{J}(V_{R})+C_{P}$$

**Figure 3:**
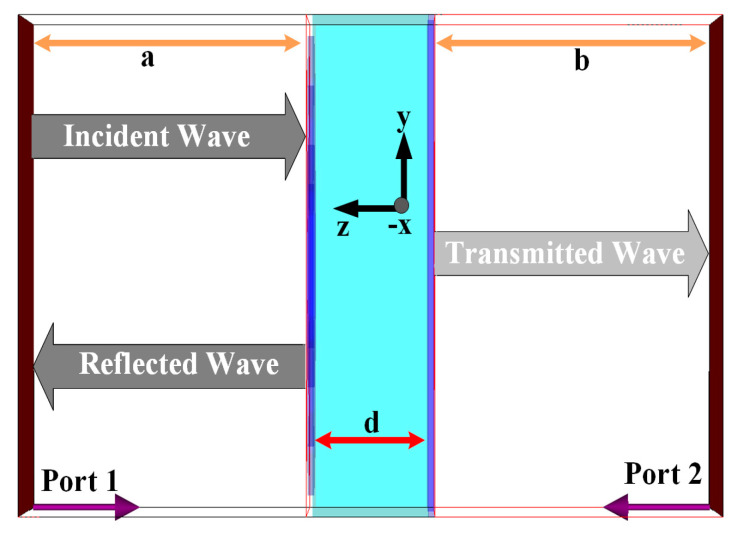
Simulation model for metamaterial parameter extraction (*d* = thickness of the slab, *a*, *b* = distance from the exciting port to reference plane).

The RTR technique was then applied in simulations and experimental validations to retrieve the effective parameters (*ε* and *µ*) of the MTM unit cell. This was performed by extracting the scattering parameters from the structure when it is excited at normal incidence [33,34]. The RTR method was chosen instead of the Nicolson–Ross–Weir (NRW) method to avoid its known phase ambiguity [28]. First, the S-parameters (i.e., reflection coefficient (*S*_11_) and the transmission coefficient (*S*_21_)) were defined within the frequency of interest (i.e., between 1 and 15 GHz). MTM simulations were performed using the model shown in Figure 3, with *d* as the thickness of the slab, and *a* and *b* are the distances from the exciting port to the reference plane. Its *S*_11_ and *S*_21_ can be mathematically calculated using Equations (3) and (4).

(3) $$S_{11}=(\frac{R_{01}(1-e^{i2nk_{0}d})}{1-R_{01}{}^{2}e^{i2nk_{0}d}})$$

(4) $$S_{21}=(\frac{(1-R_{01}{}^{2})e^{ink_{0}d}}{1-R_{01}{}^{2}e^{i2nk_{0}d}})$$

where, *k*_0_ is the wave vector in free space, *d* is the prototype/slab thickness and:

(5) $$R_{01}=\frac{z-1}{z+1}$$

The impedance (*z*) is calculated as follows:

(6) $$z=\pm \sqrt{\frac{{\left(1+S_{11}\right)}^{2}-S_{21}{}^{2}}{(1-S_{11}{}^{2})-S_{21}{}^{2}}}$$

(7) $$e^{ink_{0}d}=X\pm i\sqrt{1-X^{2}}$$

where:

(8) $$X=\frac{1}{2S_{21}(1-S_{11}{}^{2}+S_{21}{}^{2})}$$

The refractive index (*n*) is calculated as follows:

(9) $$n=\frac{1}{k_{0}d}[\{imaginary(\ln e^{ink_{0}d})+2m\pi \}-i\{real(\ln e^{ink_{0}d})\}]$$

where *m* is an integer associated with the branch index, which depends on the sinusoidal function’s periodicity. However, in this study the value of *m* was assumed to be zero [33,35,36].

Impedance (*z*) in Equation (6) and the refractive index (*n*) in Equation (9) can be calculated based on Equations (3) and (4). Furthermore, the dielectric MTM is treated as a passive medium according to field theory [33,37]. Therefore, signs in Equations (6) and (9) depend on the following conditions:

real (*z*) ≥ 0 (10)

imaginary (*n*) ≥ 0 (11)

Likewise, the permittivity (*ε*) and permeability (*µ*) are expressed as follows [34]:

(12) $$\varepsilon =\frac{n}{z}$$

(13) μ=nz

## 4. Results and Discussion

### 4.1. Modelling and Simulation

**Figure 4:**
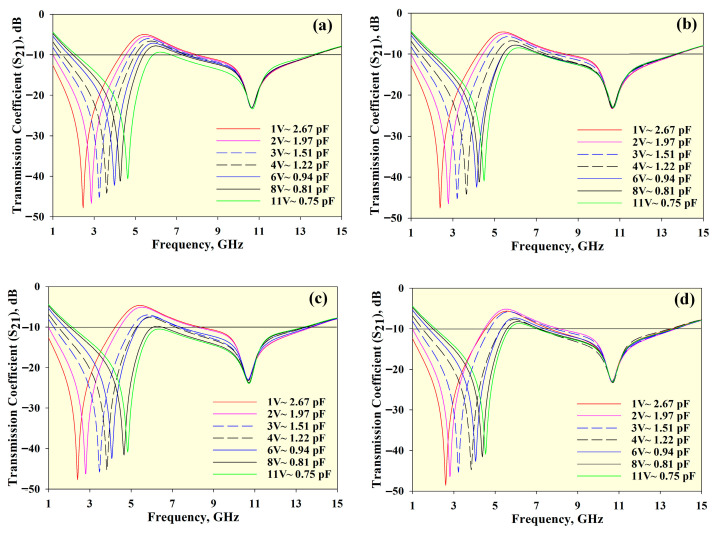
*S*_21_ results obtained from different unit cell arrays: (**a**) 1 × 1 array, (**b**) 1 × 2 array, (**c**) 2 × 1 array, and (**d**) 2 × 2 array.

The simulated *S*_21_ results of the tunable MTM for different array conditions are shown in Figure 4. Slight discrepancies can be noticed in the *S*_21_ results for all arrays, with a bandwidth of approximately 11.70 GHz. All arrays are tunable from 1 to 5.8 GHz and are nearly static from 6.9 to 13.8 GHz.

**Figure 5:**
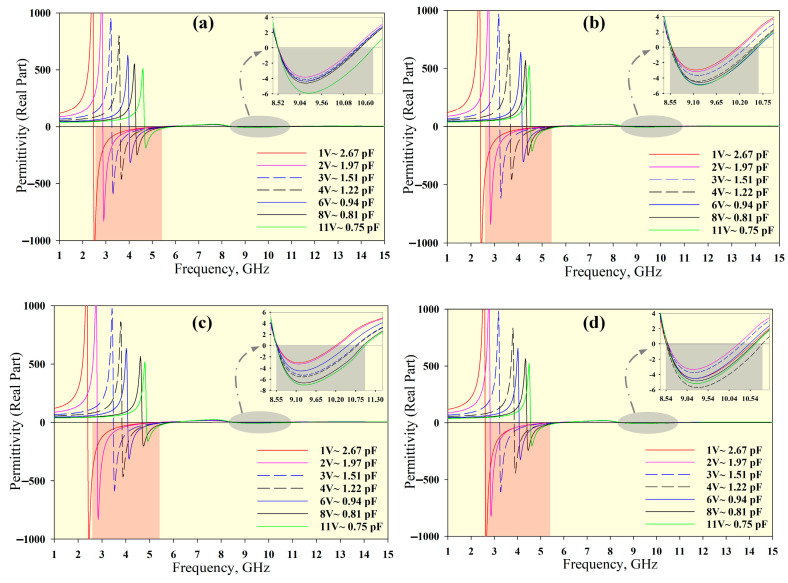
Permittivity for different unit cell arrays: (**a**) 1 × 1 array, (**b**) 1 × 2 array, (**c**) 2 × 1 array, and (**d**) 2 × 2 array.

**Figure 6:**
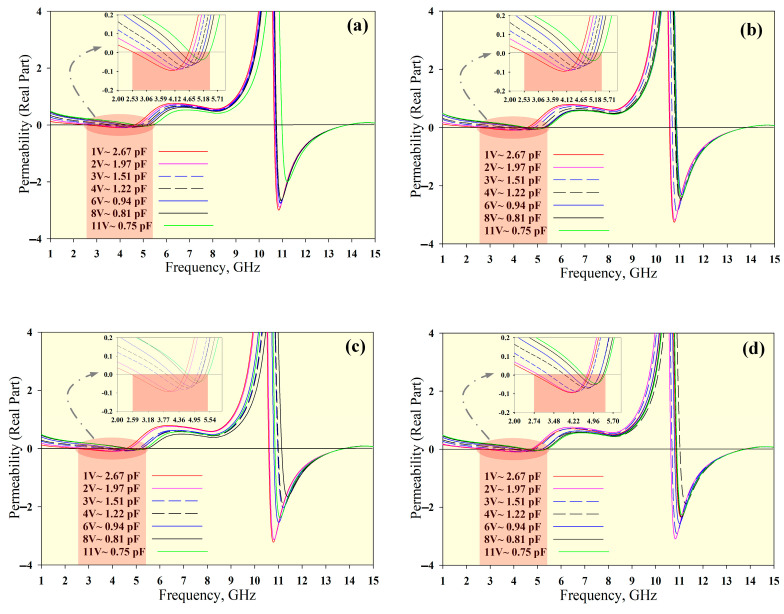
Permeability for different unit cell arrays: (**a**) 1 × 1 array, (**b**) 1 × 2 array, (**c**) 2 × 1 array, and (**d**) 2 × 2 array.

**Figure 7:**
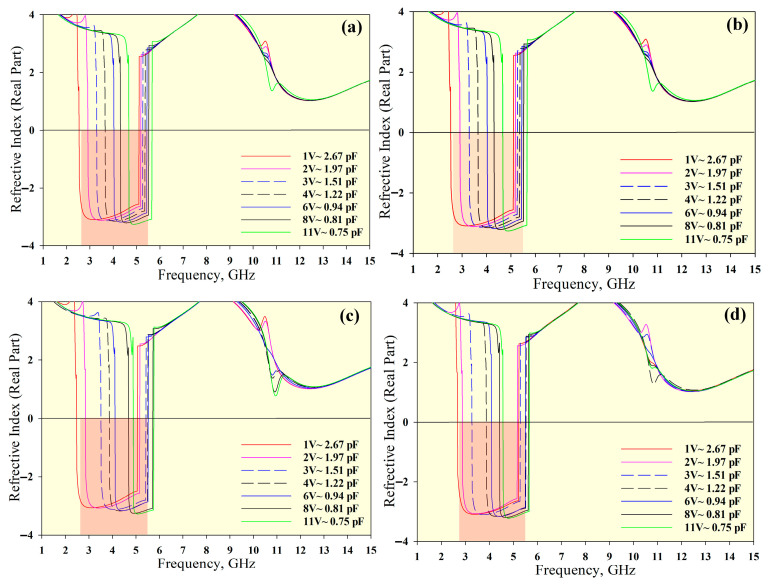
Refractive index for different unit cell arrays: (**a**) 1 × 1 array, (**b**) 1 × 2 array, (**c**) 2 × 1 array, and (**d**) 2 × 2 array.

Figure 5, Figure 6 and Figure 7 show the permittivity, permeability and refractive index, respectively, for the unit cell arrays and different tuning conditions. The results obtained using the RTR method are summarised in Table 3. The DNG region is highlighted in red in Figure 5, Figure 6 and Figure 7. The average tunable DNG bandwidth region was 2.8 GHz (from 2.58 to 5.38 GHz). Besides the DNG characteristics, the MTM also displayed ENG and MNG characteristics. The attainable average ENG BW was 3.41 GHz (from 2.46 to 5.87 GHz) and 2.28 GHz (from 8.54 to 10.82 GHz). An MNG BW of 2.8 GHz was achieved from 2.6 to 5.4 GHz, and a second band with a bandwidth of 3.18 GHz was also produced (from 10.60 to 13.78 GHz). The negative refractive index (NRI) BW was 2.87 GHz (from 2.53 to 5.40 GHz) for all unit cell arrays.

**Figure 8:**
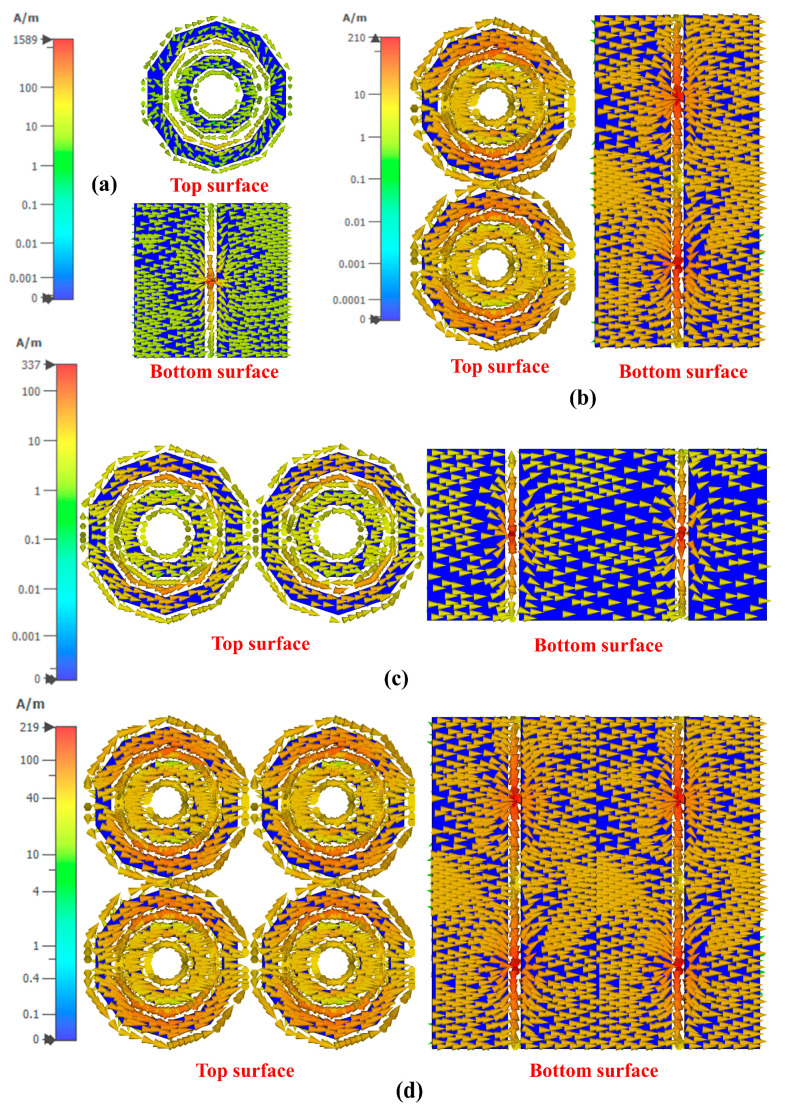
Surface current distribution of different unit cell array conditions: (**a**) 1 × 1, (**b**) 1 × 2, (**c**) 2 × 1, and (**d**) 2 × 2 arrays.

Figure 8 illustrates the simulated surface current distribution in the *xy*-plane at 5 GHz (with an applied reverse bias voltage of 11 V~0.75pF). This frequency was selected in the DNG region of the proposed MTM. The arrows indicate the current paths, whereas the colour expresses the current intensity. The currents around the inner edges of the MTM are the highest compared to other areas. A series of simulations was then performed where the capacitance was tuned to study the effects of the varactor diode on the MTM. Two forms of currents exist: conduction currents and displacement currents. The conduction current exists on the top and bottom layers, whereas the displacement current flows between these layers. Strong surface current distribution could still be observed when the MTM was tuned to other frequencies within the average tunable range (from 2.53 GHz to 5.4 GHz).

### 4.2. Deformation Analysis

**Figure 9:**
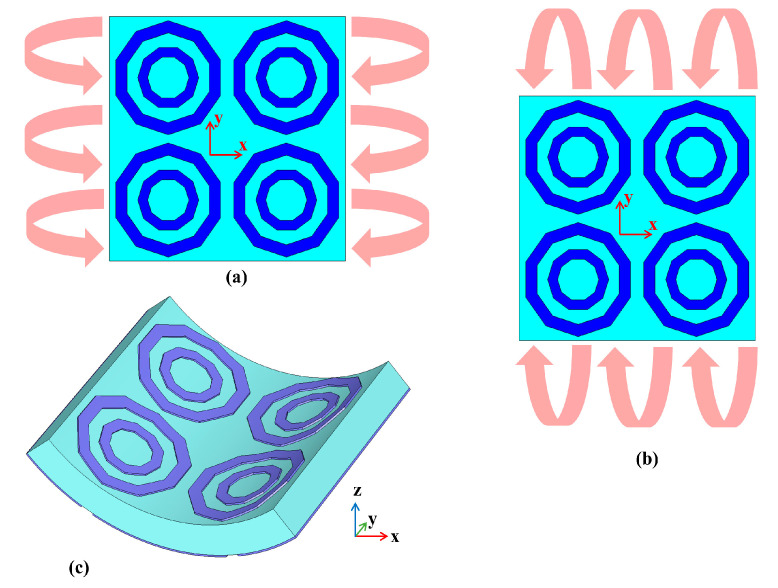
Possible bending at different radii at (**a**) *x*-axis and (**b**) *y*-axis. (**c**) An example of deformation where the 3D structure of the MTM is changed.

The MTM structures are unique as they are designed on flexible materials. Due to this, the performance of the 2 × 2 MTM unit cell array was further analysed under deformed (different bending) conditions commonly observed in clothing. These deformations are typically caused by human body morphology and movements. Figure 9 depicts the possible physical deformations of the MTMs’ (elongation or bending) resulting in the changes in the EM properties of MTM [23].

**Figure 10:**
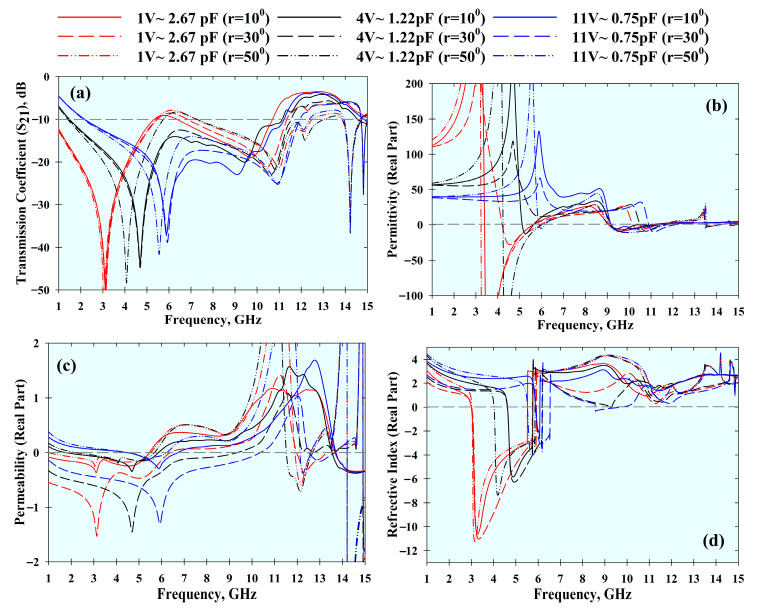
Effects of bending at different radii (*r*) at the *x*-axis with different tuning conditions: (**a**) *S*_21_ result, (**b**) permittivity, (**c**) permeability, and (**d**) refractive index.

**Figure 11:**
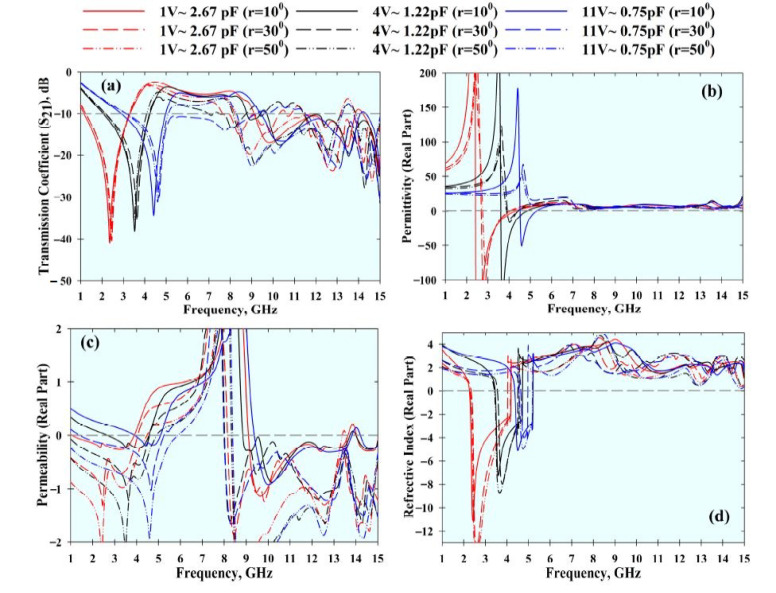
Effects of bending at different radii (*r*) at the *y*-axis with different tuning conditions: (**a**) *S*_21_ result, (**b**) permittivity, (**c**) permeability, and (**d**) refractive index.

The effective parameters and the *S*_21_ result of the MTM under distinctive bending radii (*r*) were studied. In general, this results in resonant frequency shifts, as shown in Figure 10 and Figure 11. Nonetheless, the MTM still exhibited left-handed characteristics with different bending conditions and varactor tuning states.

### 4.3. Experimental Validations

**Figure 12:**
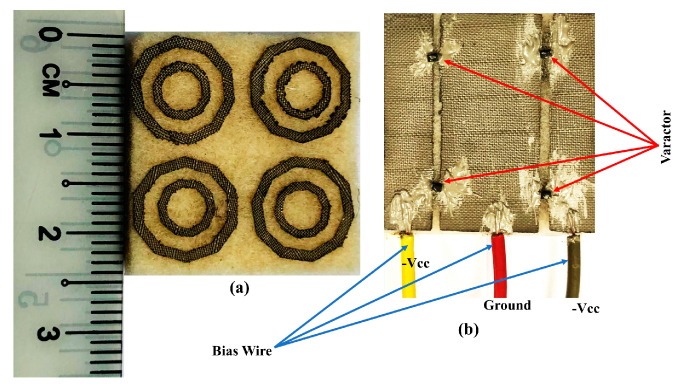
Fabricated 2 × 2 unit cells array (24 × 24 mm^2^) proposed structure. (**a**) Front view. (**b**) Back view.

**Figure 13:**
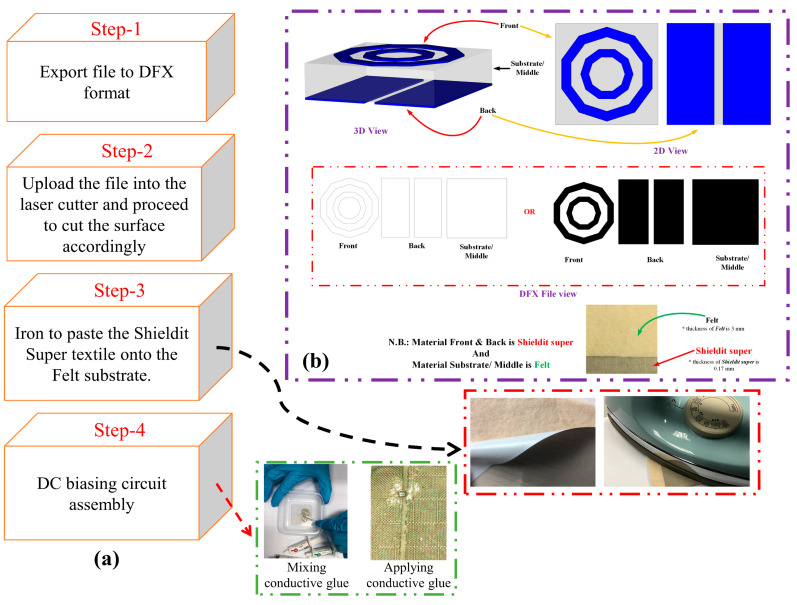
(**a**) Flowchart of the fabrication steps and procedure. (**b**) Graphical illustration for the laser cutting service provider.

Figure 12 shows the fabricated prototype used for the experimental validations, which were performed using a laser cutter to ensure dimensioning accuracy. The overall process is depicted in Figure 10a. First, the final prototype’s outline was exported into the Drawing Exchange Format (DFX) file. This DFX file was then inserted into the laser cutter and used to dimension the ShieldIt Super^TM^ textile, as shown in Figure 13b. Next, a clothing iron was used to heat the dimensioned ShieldIt textile to attach it onto the felt substrate. The ShieldIt textile was secured onto the felt substrate using the available layer of heat-melt glue behind ShieldIt. This was followed by the assembly of the DC biasing components. These components (i.e., RF varactor diodes and wires) were connected to the textile using a conductive epoxy from CircuitWorks^®^, Kennesaw, Georgia, USA [38]. Prior to this, the epoxy was thoroughly mixed with the hardener at a 1:1 ratio for two minutes. Its application onto the ShieldIt surface was completed within 8 min. Finally, the epoxy connections to the textile’s surface were cured for between 24 and 36 h above 25 °C.

**Figure 14:**
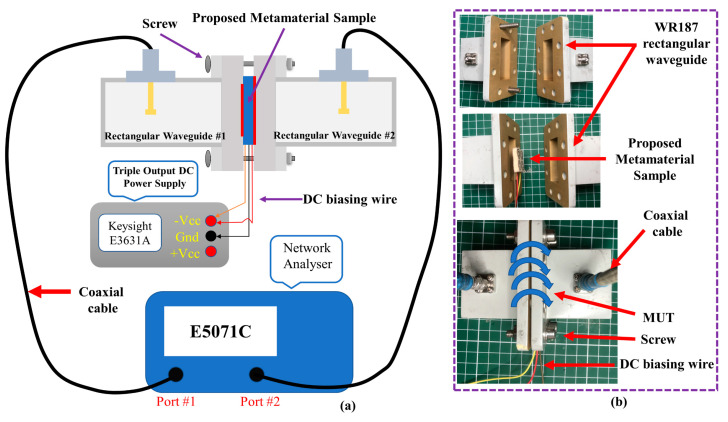
Measurement setup. (**a**) Graphical representation of the measurement procedure, and (**b**) photos of the material under test (MUT) positioned between the waveguides (WGs) (where WR187 WG is dimensioned at 47.5488 × 22.1488 mm^2^).

Measurements were then performed using an E5071C network analyser (Agilent Technologies, Bayan Lepas, Penang, Malaysia) to verify the simulated results. The MTM was evaluated using different sets of standard rectangular waveguides (WGs) to avoid the use of custom sample holders [28]. Due to the wide tunability range of the MTM, four different types of WG were used: WR430, which operates between 1.7 and 2.6 GHz; WR284, which operates between 2.6 and 3.95 GHz; WR187, which works between 3.95 and 5.85 GHz; and WR137, which works between 5.85 and 8.20 GHz. Measurements were performed with the MTM placed in between the cross-sections of the WGs, and the reverse bias voltage was supplied using an E3631A power supply (Keysight Technologies, Bayan Lepas, Penang, Malaysia), as depicted in Figure 14. The coaxial cables were first calibrated using the standard short-open-load-transmission (SOLT) procedure prior to the calibration of the WGs using the through-reflect-line (TRL) method [28,39]. Once measurements were completed for the MTMs, all scattering data were combined within the frequency range of interest (1.7 to 8.2 GHz). Finally, the parameters of the MTM were retrieved using the RTR method described previously.

**Figure 15:**
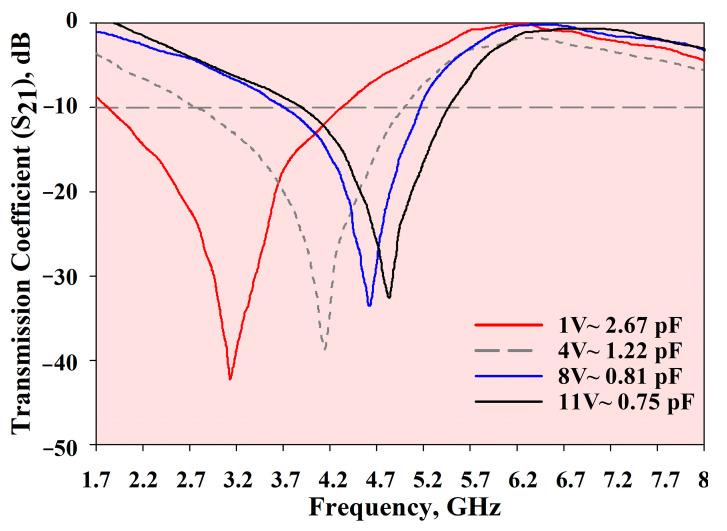
*S*_21_ measurement results.

**Figure 16:**
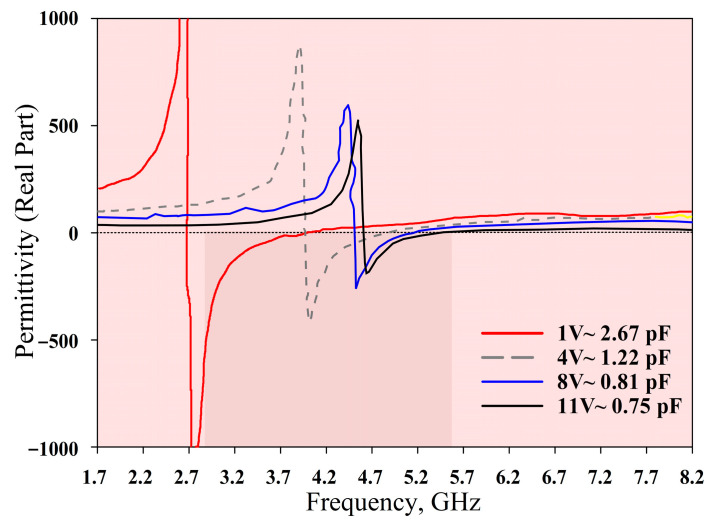
Permittivity measurement results.

**Figure 17:**
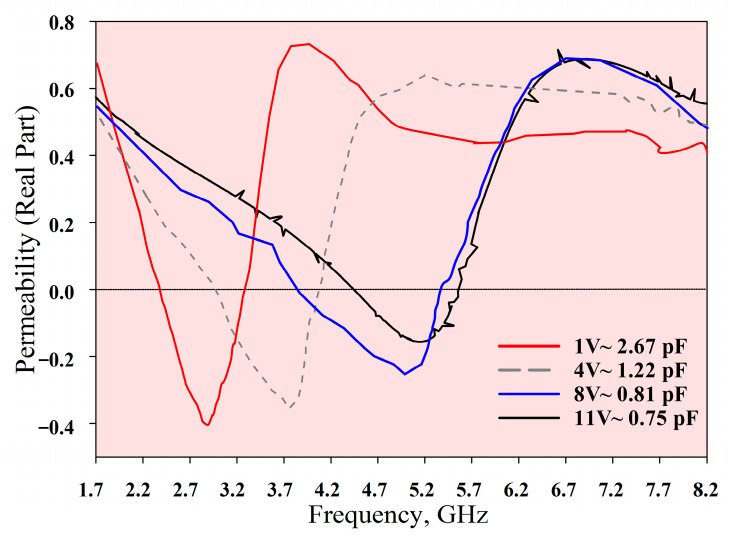
Permeability measurement results.

**Figure 18:**
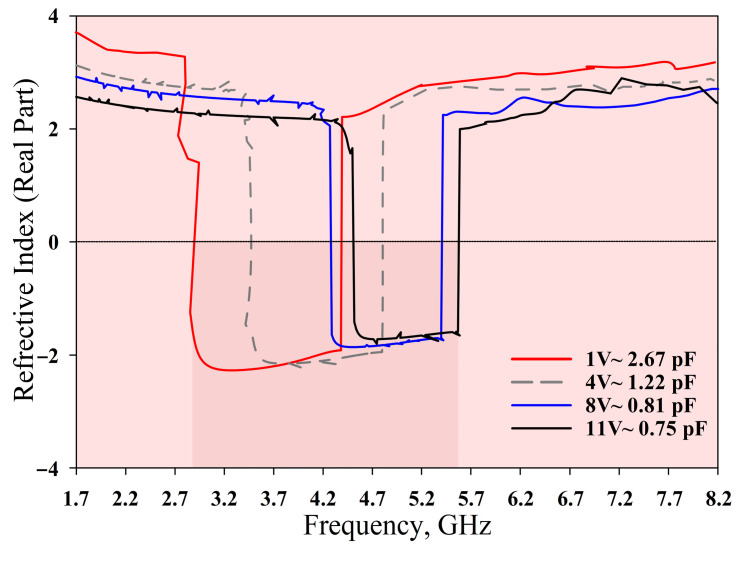
Refractive index measurement results.

The measured *S*_21_, *ε*, *µ*, and refractive index of the MTM are depicted in Figure 15, Figure 16, Figure 17 and Figure 18, respectively. The experimental results show that the tunable *S*_21_ BW was 3.91 GHz, from 1.77 to 5.68 GHz, and this agrees with simulations where the tunable BW was 4.1 GHz (from 1.7 to 5.8 GHz). On the other hand, the measured ENG BW was 3.04 GHz, from 2.69 to 5.73 GHz, whereas simulations indicated that the ENG BW was 3.24 GHz (from 2.60 to 5.84 GHz). Similarly, the measured MNG BW was 3.2 GHz (from 2.36 to 5.56 GHz) whereas simulated MNG BW was from 2.74 to 5.39 GHz. Finally, the NRI BW of 2.76 GHz was obtained from measurements (from 2.75 to 5.51 GHz), whereas simulations indicated an NRI BW of 2.89 GHz (from 2.67 to 5.65 GHz). In the measurements, the achievable overall tunable DNG region was from 2.75 to 5.51 GHz, as highlighted in grey. All results between 1.7 GHz and 8.2 GHz are summarised in Table 4. In general, all measured BW were in good agreement with the simulations, with slight discrepancies. Furthermore, as shown in Table 4, the *S*_21_ BW was reduced by 190 MHz whereas the starting frequency also shifted from 1.7 GHz to 1.77 GHz. Again, the ENG BW was decreased by 200 MHz and the result was slightly shifted to a higher starting frequency by 70 MHz. On the contrary, the measured MNG BW was increased by 460 MHz compared to the simulations, and the measurements indicated the shifting of the starting frequency to a lower frequency by 380 MHz. However, the measured NRI BW was decreased by 130 MHz while the starting frequency was shifted to a higher frequency by 80 MHz compared to the simulations.

## 5. Conclusions

This paper presents an electrically tunable, compact, and flexible MTM designed using a combination of decagonal-shaped SRR and a slotted ground plane loaded with a RF varactor. The MTM parameters were successfully tuned using four RF varactor diodes deployed at the slotted ground plane of the 2 × 2 MTM array. Simulations and experimental results showed good agreement, validating the tunability of the MTM. The results also indicated that the DNG characteristics are tunable within the S and C bands, whereas the ENG and MNG characteristics are tunable within the X-band. It can be concluded that the proposed MTM can be effectively tuned to facilitate future wearable applications in a flexible/wearable format.

## Figures and Tables

**Table 1:** Comparison of this work with other tunable metamaterials (MTMs).

Reference	Method/Component	Substrate Type & Thickness	Varactor Tuning Mechanism	Tunable Parameters	Tuning Ratio (%)
[29]	Varactor	FR3-epoxy (rigid) & 1.4 mm	External DC power	Reflection coefficient phase	3.72
[17]	Varactor	RT/Duroid (rigid) & 0.8 mm	–	Medium index/ refractive index	3.34
[19]	Varactor	FR-4 (rigid) & 1.6 mm	–	Absorption range	9.24
[15]	Varactor and photodiode	FR-4 (rigid) & 1.5 mm	Light intensity control using photodiodes.	MNG properties/ artificial magnon resonance	–
[30]	Varactor	Rogers R4003 (rigid) & 0.5 mm	Varying the amplitude of the propagating EM waves	Magnetic resonance and transmission coefficient	–
This work	Varactor	Felt (Flexible) & 3 mm	External DC power	Transmission coefficient (*S*_21_), ENG, MNG, NRI and DNG properties	*S*_21_ = 104.97, ENG = 72.21, MNG = 80.81, NRI = 66.83, and DNG = 66.83

**Table 2:** Parameters of the MTM unit cell.

Parameters	*W*	*L*	*R*_1_	*R*_2_	*R*_3_	*R*_4_	*G*_1_	*h*	*t*
**Value (mm)**	12	12	5.6	4.4	3.1	2.1	1	3	0.17

**Table 3:** ENG, MNG, NRI and DNG bandwidths for different unit cell arrays for different varactor tuning conditions.

Array Structure	ENG BW (GHz)	MNG BW (GHz)	NRI BW (GHz)	DNG BW (GHz)
1 × 1	2.46–5.86 & 8.52–10.8	2.54–5.40 & 10.64–13.77	2.53–5.66	2.54–5.40
1 × 2	2.40–5.84 & 8.55–10.64	2.53–5.40 & 10.57–13.78	2.46–5.65	2.53–5.40
2 × 1	2.40–5.96 & 8.55–11.00	2.59–5.41 & 10.59–13.78	2.47–5.76	2.59–5.41
2 × 2	2.60–5.84 & 8.54–10.83	2.74–5.39 & 10.63–13.79	2.67–5.65	2.74–5.39

BW = Bandwidth, ENG = Epsilon Negative, MNG = mu Negative, NRI = Negative Refractive Index, DNG = Double Negative.

**Table 4:** Simulated and measured *S*_21_, ENG, MNG, NRI and DNG bandwidths between 1.7 GHz and 8.2 GHz for a 2 × 2 unit cell array for different varactor tuning conditions.

Array Structure	*S*_21_ BW (GHz)	ENG BW (GHz)	MNG BW (GHz)	NRI BW (GHz)	DNG BW (GHz)
Simulated	1.7–5.80	2.60–5.84	2.74–5.39	2.67–5.65	2.74–5.39
Measured	1.77–5.68	2.69–5.73	2.36–5.56	2.75–5.51	2.75–5.51

## Data Availability

The study did not report any data.
